# Supplementary figures and images for: Label-Free Quantitative Acetylome Analysis Reveals Toxoplasma gondii Genotype-Specific Acetylomic Signatures
Source: Microorganisms. 2019 Oct 30;7(11):510. doi: 10.3390/microorganisms7110510 (PMC6921067; doi:10.3390/microorganisms7110510)

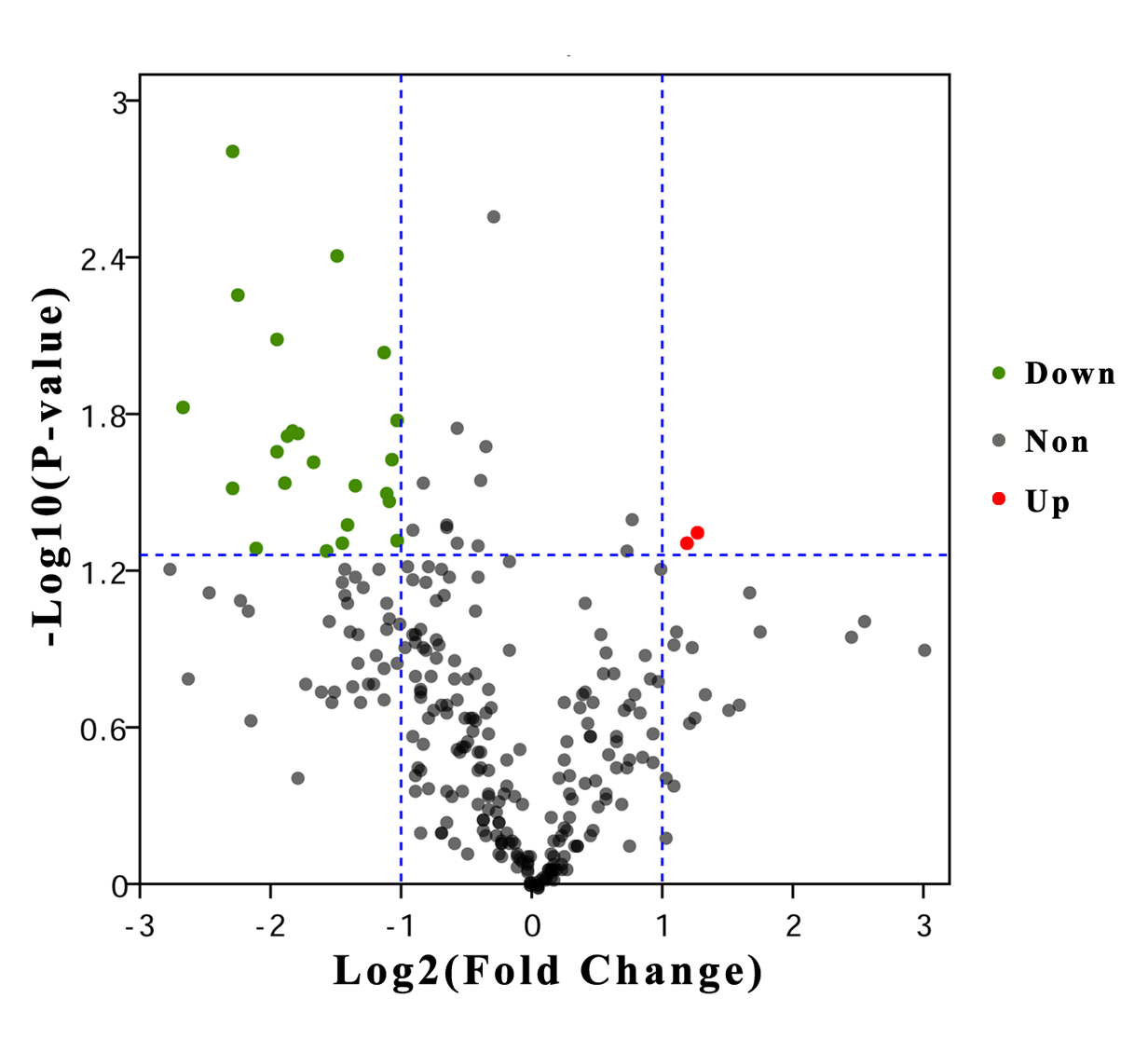

Supplement: Supplementary file 1 [file microorganisms-07-00510-s001.zip › microorganisms-615490-SI/Figure S1.tif]

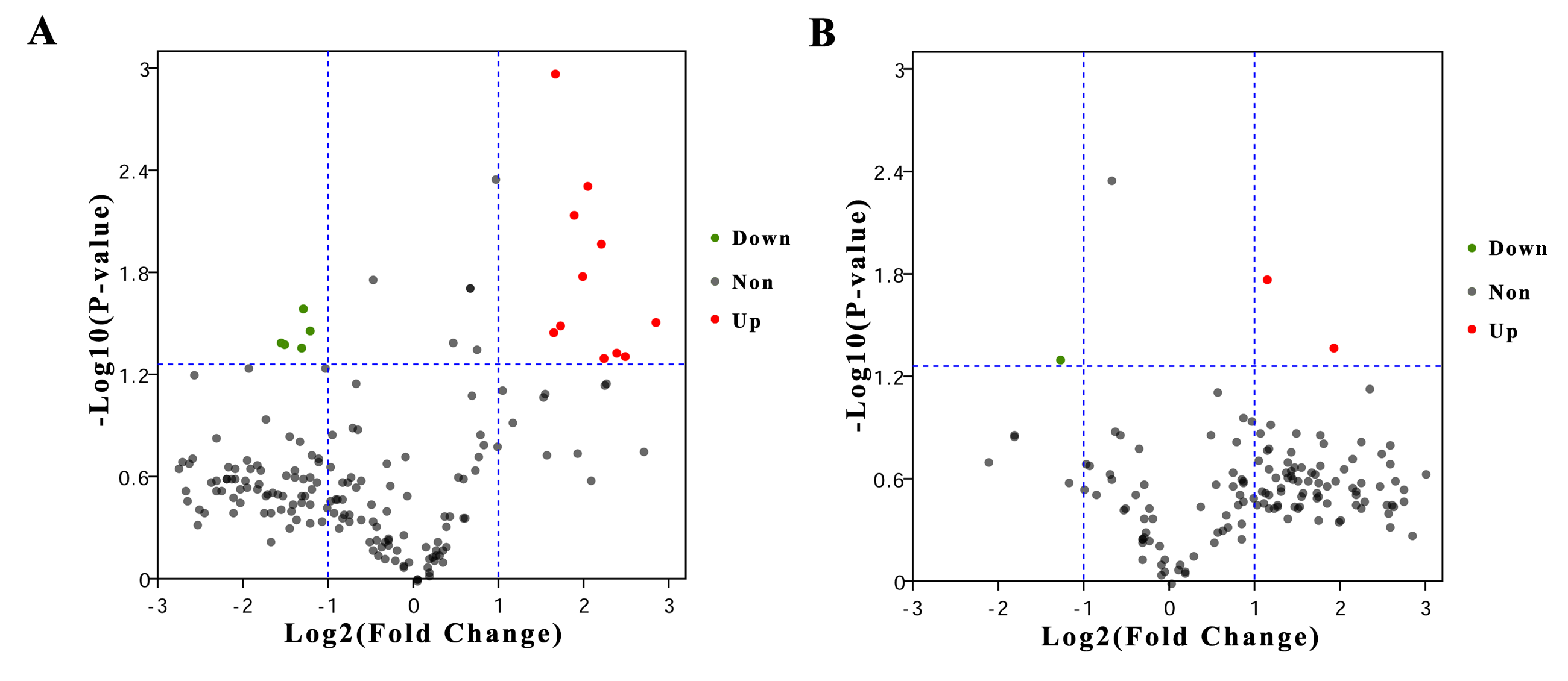

Supplement: Supplementary file 1 [file microorganisms-07-00510-s001.zip › microorganisms-615490-SI/Figure S2.tif]

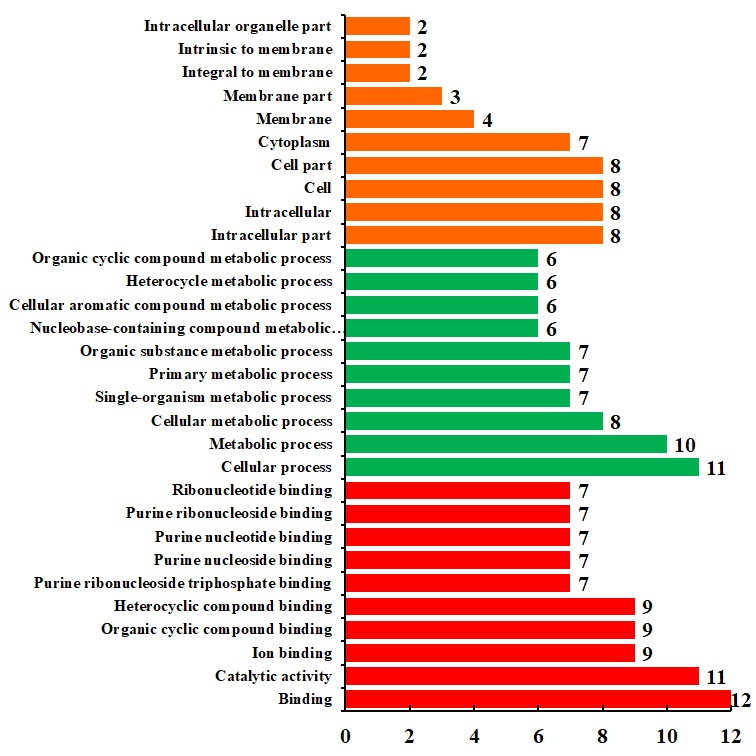

Supplement: Supplementary file 1 [file microorganisms-07-00510-s001.zip › microorganisms-615490-SI/Figure S3.jpg]

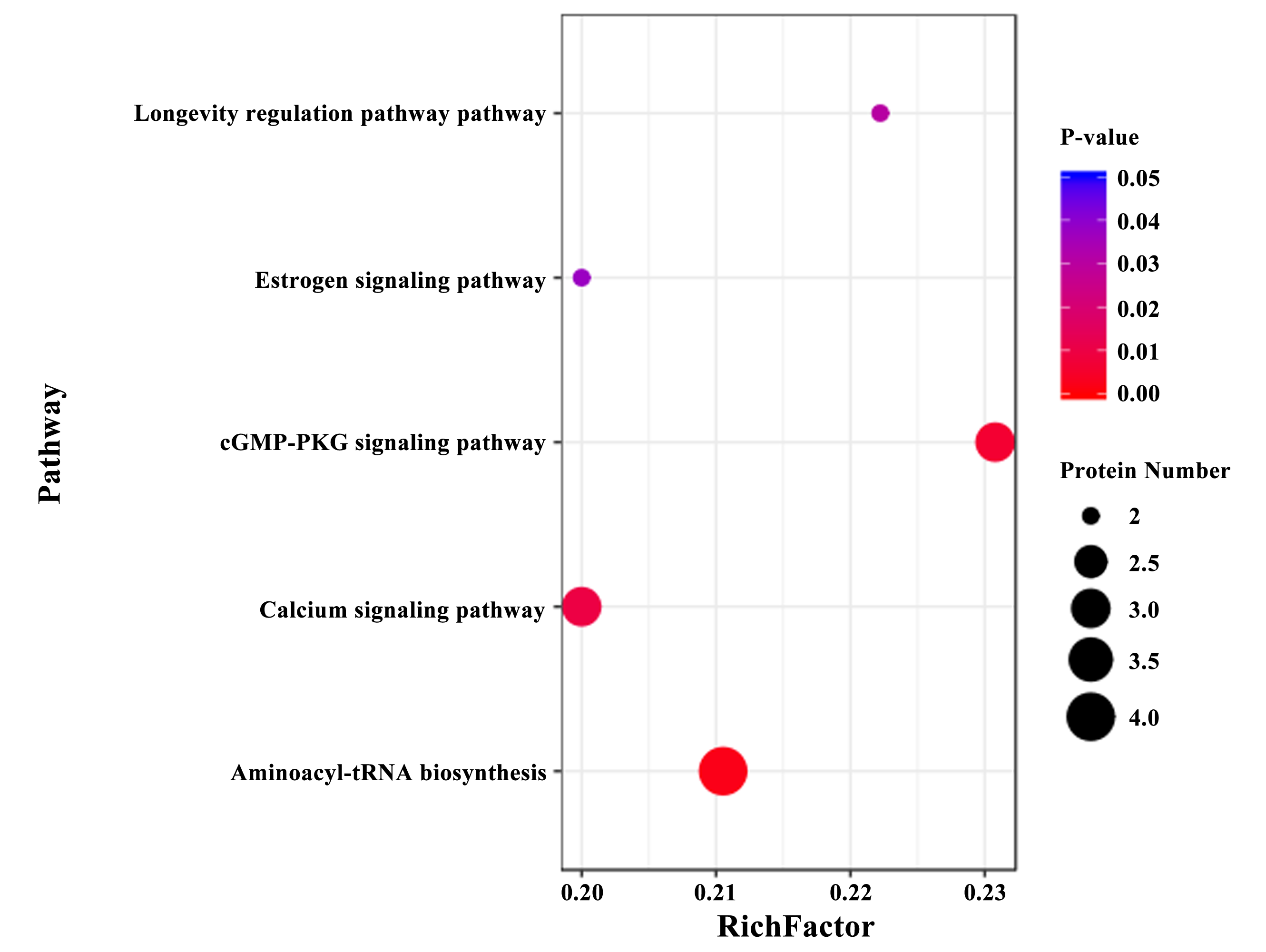

Supplement: Supplementary file 1 [file microorganisms-07-00510-s001.zip › microorganisms-615490-SI/Figure S4.tif]

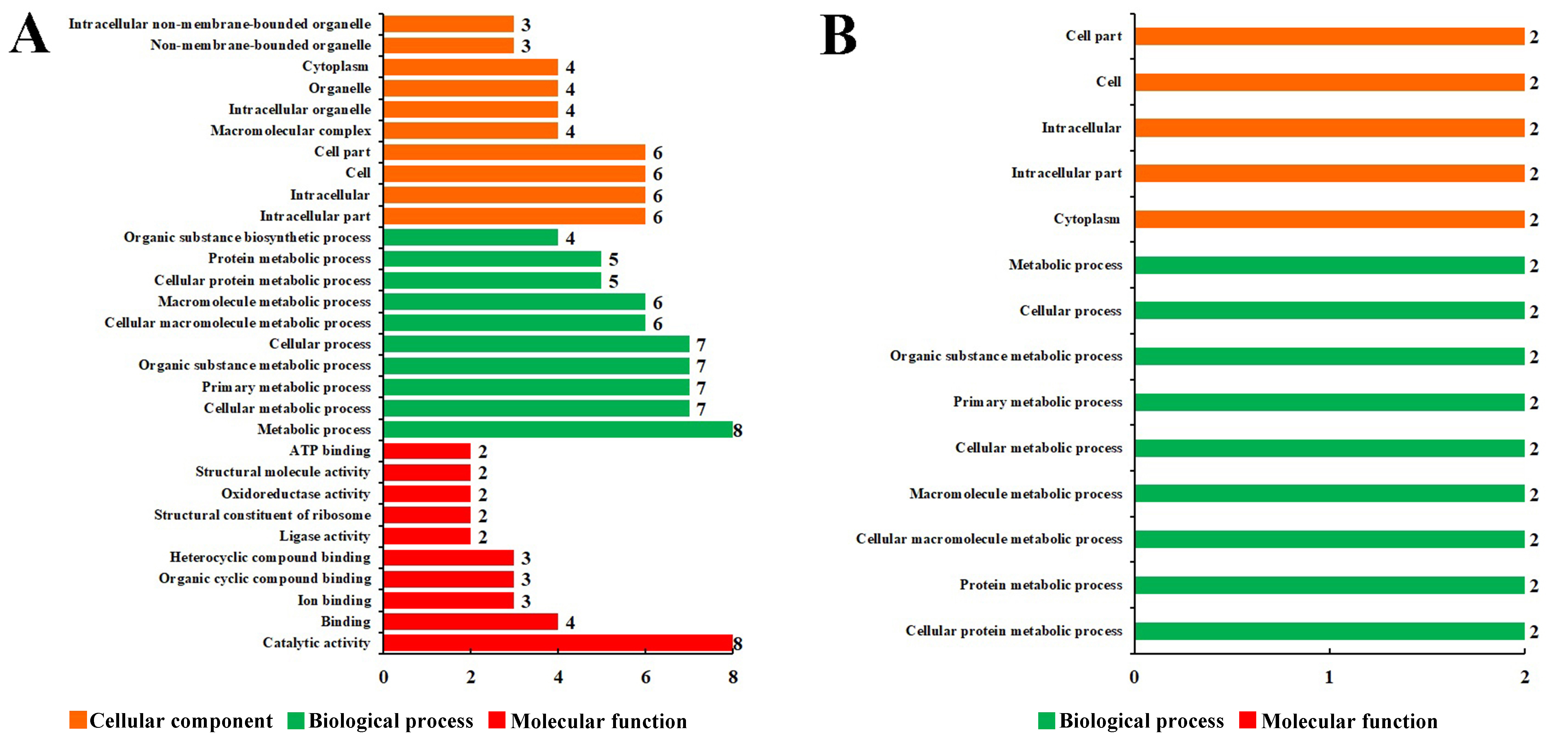

Supplement: Supplementary file 1 [file microorganisms-07-00510-s001.zip › microorganisms-615490-SI/Figure S5.jpg]

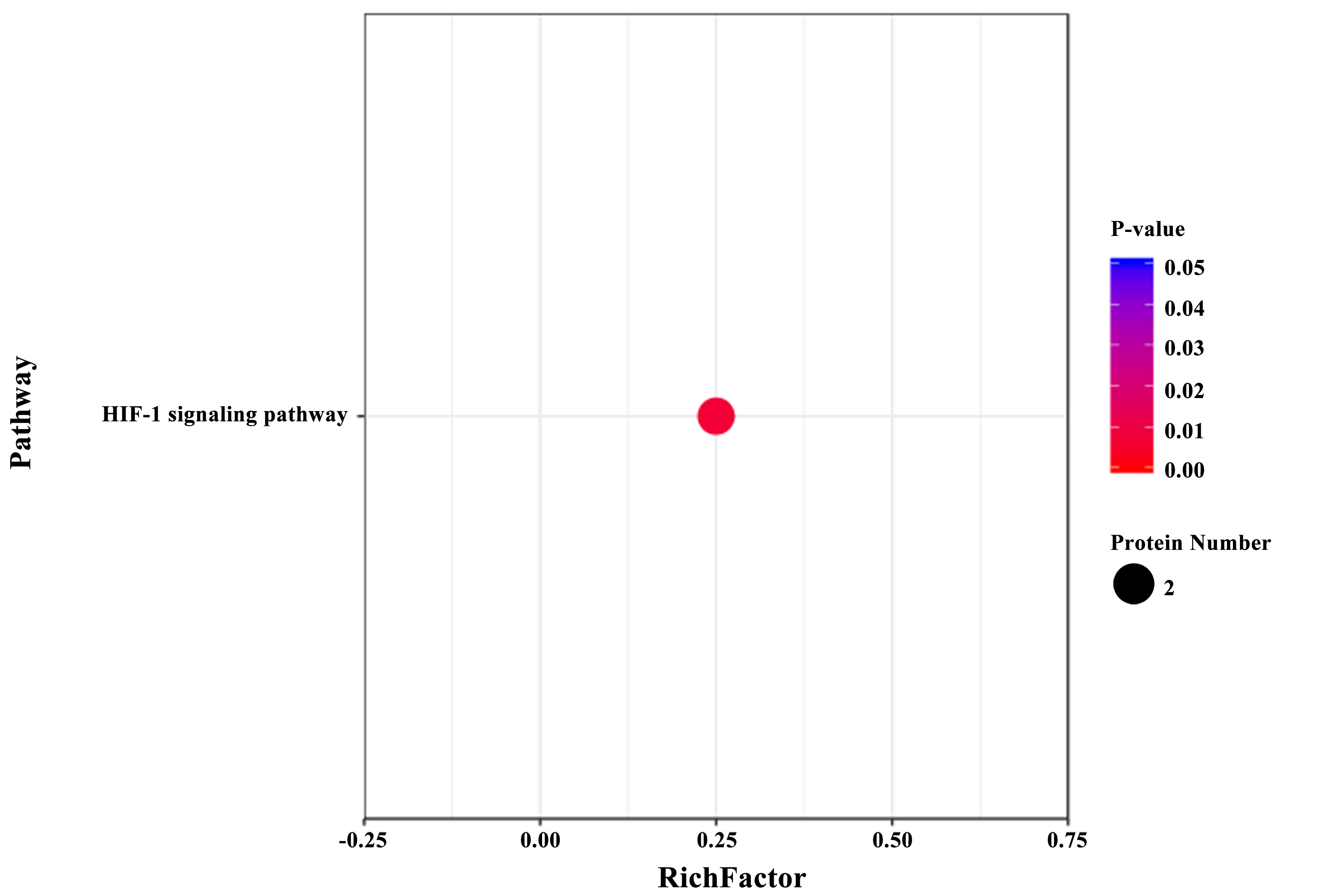

Supplement: Supplementary file 1 [file microorganisms-07-00510-s001.zip › microorganisms-615490-SI/Figure S6.tif]
